# Fibrolipoma of the nasal septum; report of the first case

**DOI:** 10.1186/1916-0216-42-11

**Published:** 2013-02-02

**Authors:** Murat Ozturk, Kadri Ila, Ahmet Kara, Mete Iseri

**Affiliations:** 1 Department of Otorhinolaryngology, Kocaeli University Medical Faculty, Kocaeli, Turkey; 2 KocaeliUniversitesi Tip Fakultesi, KBB Anabilim Dali, Umuttepe, Kocaeli, 41380, Turkey

**Keywords:** Nasal obstruction, Lipoma, Nasal septum

## Abstract

**Background/Objective:**

Fibrolipomas are a rare subtype of lipomas and very rare in the oral and maxillofacial region. Lipomas affecting the central nervous system are even more infrequent occurring with a frequency of 0.1%.

**Study design, methods:**

Case report.

**Case presentation:**

This report includes a patient who had a nasal septal fibrolipoma and an accompanying corpus callosum lipoma.

**Conclusions:**

To our knowledge, this is the first reported nasal septal fibrolipoma case in the literature. The diagnostic and surgical features of this case and the unity of septal fibrolipoma and intracranial lipomas are discussed.

## Background

Lipomas are benign, slowly growing neoplasms composed of mature fat cells grouped in lobules by connective tissue septa. These tumors are very rare in the sinonasal tract [1]. Fibrolipomas are a very rare subtype of the lipomas, composing %1,6 of the facial lipomas [2]. In the literature; there are cases reported as fibrolipomas in the esophagus, pharynx, colon, trachea, larynx and oral cavity [3]. This article presents a patient with nasal septal fibrolipoma, and to the knowledge of the researchers, it is the first reported nasal septal case in the literature.

## Case presentation

**Figure 1:**
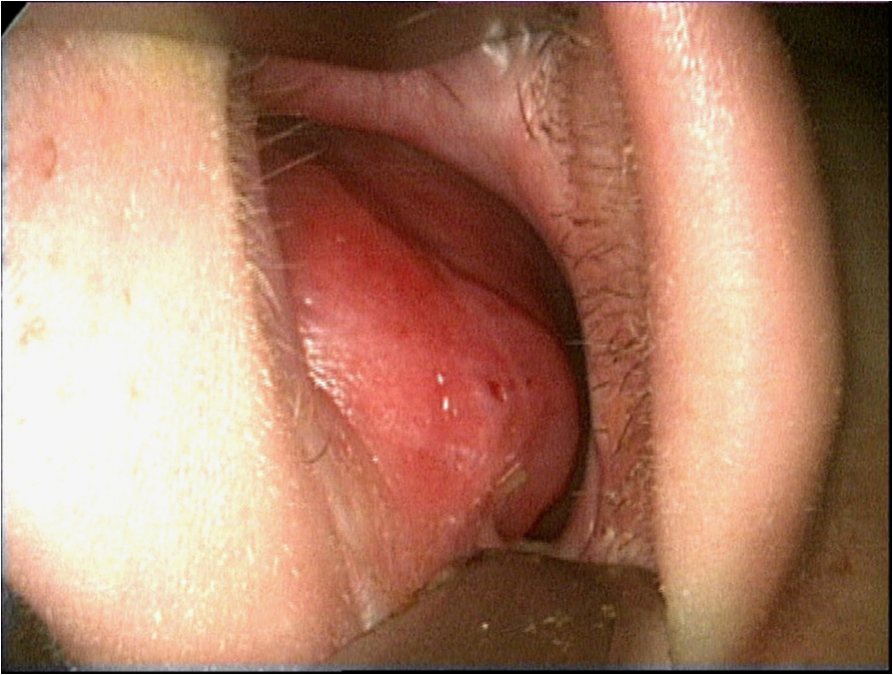
Soft, smooth mass with normal nasal mucosa in the left nasal cavity.

**Figure 2:**
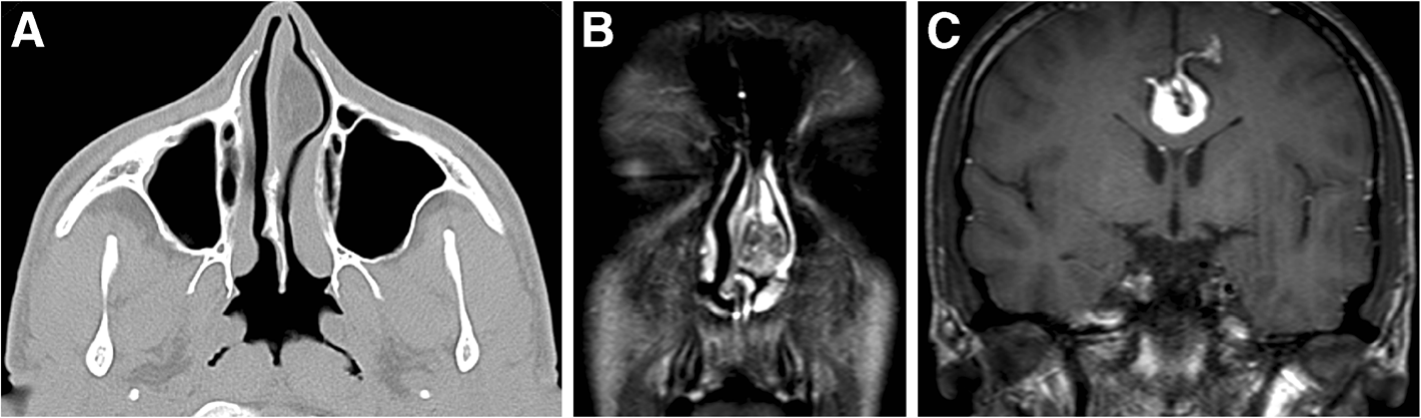
**(A) Computed tomography showing a 25x12 mm low density mass in the nasal septum.** (**B**) T2 weight magnetic resonance (MR) imaging presenting a high signal intensity 22x12 mm mass. (**C**) MRI showing a lipoma, 18 mm thick and 51 mm in length at the level of falx cerebri that pushed the corpus callosum and reaching the cingulate gyrus anteroposteriorly.

**Figure 3:**
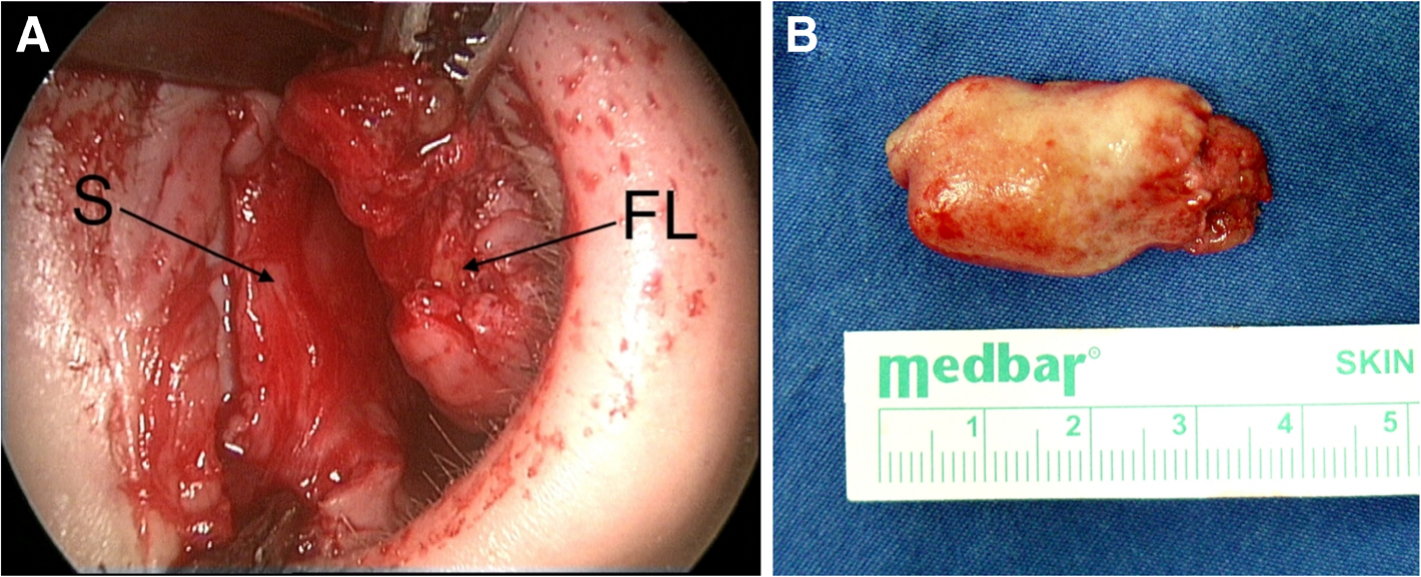
**(A) The medial dissection plan between the mass and submucosal tissues and perichondrium (S: septum, FL: fibrolipoma).** (**B**) The macroscopic view of the excised mass.

**Figure 4:**
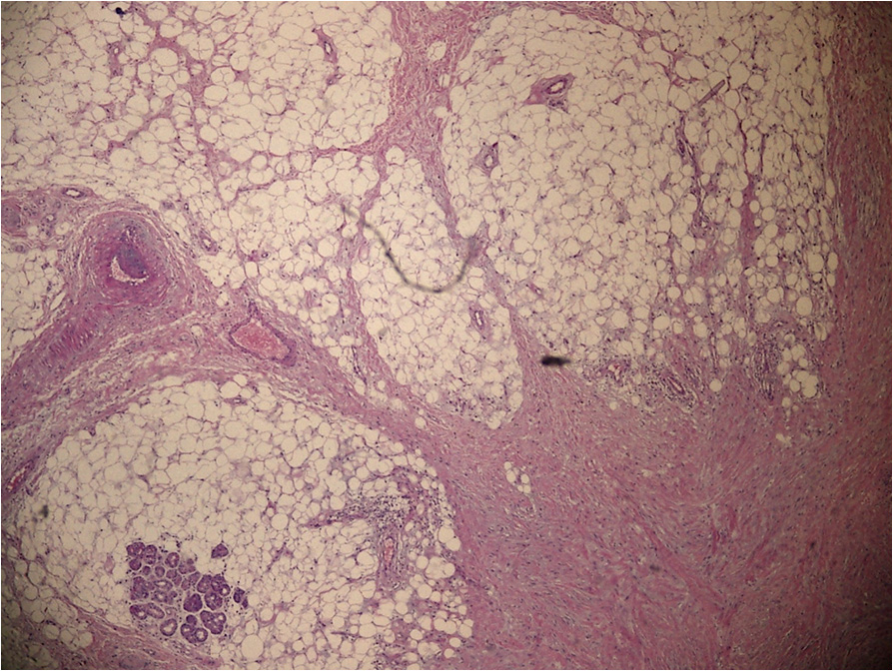
The histopathologic view under light microscope (H&E, x40); the section shows massive mature adipocytes with proliferation of the neighboring fibrous tissue.

An 18-year-old male patient presented with a two year history of nasal obstruction to the otorhinolaryngology department with no additional complaints. His medical family history had revealed nothing abnormal. An endoscopic examination revealed a soft, painless, well circumscribed 2.5x1.5 cm sized mass coated with normal mucosa in the left nasal cavity, localized from the anterior portion of the nasal septum to the posterior (Figure 1). The right nasal cavity was deemed narrowed due to the compression effect of the mass. The computed tomography (CT) showed a 25x12 mm sized low density mass in the septum (Figure 2A). Magnetic resonance imaging (MRI) presented a 22x12 mm lesion with high signal intensity in both T1 and T2 weighted images (Figure 2B). The image revealed a suppression pattern on the fat-suppressed MRI. The same investigation also revealed a 18 mm thick, 51 mm long lipoma at the falx cerebri that pushed the corpus callosum and anteroposteriorly reached the cingulate gyrus (Figure 2C). An incisional biopsy was performed on the mass under local anesthesia, and the specimen was reported as fibrolipoma. This was followed by a surgical excision under general anesthesia. The operation began with a hemitransfixion incision, and continued with the elevation of the septal mucosa with a Freer elevator to the mass. The mass was medially dissected from the perichondrium and submucosal tissues easily, but it was very difficult to dissect it from the lateral submucosal tissues. The capsule of the mass was very adhesive at lateral side and a dissection plan between the capsule and submucosal tissues could not be found (Figure 3A). A 2.5x2 cm sized soft mass was excised with the septal mucosa lateral to the fibrolipoma, and the medial submucosal tissues and perichondrium remained intact (Figure 3B). A bilateral silicon nasal tampon was inserted into the nasal cavity. There were no postoperative complications. The final histopathologic examination of the excised mass confirmed the preoperative diagnosis of fibrolipoma (Figure 4). The patient consulted the neurosurgery department for the intracranial lipoma, it was suggested that the patient return upon experiencing any symptoms such as seizure, dementia, heightened intracranial pressure, or local neurogenic symptoms.

## Conclusions

Lipomas are well circumscribed, slow growing, benign tumors composed of mature fat cells, of which 13% arise in the head and neck region [1]. The peak incidence is in the fifth and sixth decades of life, and they are rare under the age of 20 years [4]. Lipomas are divided as simple lipoma and variants such as: fibrolipoma, angiolipoma, chondroid lipoma, myolipoma, spindle cell/pleomorphic lipoma, diffuse lipomatous proliferations (lipomatosis) and hipernoma. Lipomas can develop in various parts of the body, but a lipoma occurring in the nasal cavity is an extremely rare entity, and there are few cases reported in the literature [4]. Most cases are under the age of 1 year. There was only one adult reported a nasal septal lipoma [4].

Fibrolipomas are classified as a variant of lipomas by the World Health Organization (WHO) and they are very rare in the oral and maxillofacial regions. Both lipomas and fibrolipomas are well circumscribed and have a thin capsule. Fibrolipoma differs from the classic variants because the mature adipose tissue is interspersed by bands of connective tissue [3]. Additionally, fibrolipomas have a higher proliferative activity than the classic variants [5]. Although fibrolipomas are known as benign tumors, there are a few cases of conversion to liposarcoma in the literature [2].

Fibrolipomas occur in the subcutaneous tissue, oral cavity, esophagus, pharynx, colon, trachea, larynx, parotid gland, and the spermatic cord as a slow growing mass [2,3]. However, development in the nasal septum has not yet to be reported. The age of the patient in this case was also unusual, because these lesions’ peak incidence is in the fifth and sixth decades. They are rarely observed under the age of 20 years.

MRI is very useful for the diagnosis of all kind lipomas. Tumors have high signal intensity on T1-weighted images, with relative decreasing signal on T2-weighted images [6]. A fat-suppressed MRI is particularly beneficial for diagnosis. On the other hand, fibrolipomas are more heterogeneous than lipomas on MRI images. This was a cause of concern in the study patient’s preoperative diagnosis. Our radiologists could not precisely diagnose the mass as a lipoma. The MRI images were atypical and did not give a specific pre-diagnosis. In addition, there were no cases of nasal septal fibrolipoma in the literature, which could lead to a diagnosis. On CT images, lipomas can be seen as an uncontrasted hypodense mass, as demonstrated in the image of the subject.

On the other hand, lipomas affecting the central nervous system are even more infrequent occurring with a frequency of 0.1%, with most localized in the corpus callosum. Although most are asymptomatic, some present with seizure, dementia, raised intracranial pressure, and hemiparesis. Surgical resection of a callosal lipoma is technically difficult because of the close association with anterior cerebral arteries and the adherence of the capsule to the adjacent brain. If seizures occur because of the callosal lipomas, anti-convulsants can be prescribed [7]. The current subject’s corpus callosum lipoma was incidentally recognized during the examination of the nasal mass MRI. The patient has no complaints related to this lipoma, and did not report seizures or dementia. The relationship between corpus callosum lipoma and nasal septal lipoma is interesting and should be kept in mind.

It is very rare to observeboth nasal lipoma and intracranial lipoma in the same patient. In the literature, there are a few cases presented as a rare entity called Pai syndrome, consisting of a midline cleft of the upper lip, facial skin polyps and central nervous system lipomas [8]. Hollis reported two patients with nasal lipoma accompanied by pericallosal lipoma [7]. Both were newborns and their skin-covered nasal masses protruded through the nostril. The first patient had two other swellings, one on the forehead and the other in the upper gingival sulcus, and the other patient had two associated abnormalities, namely a notched upper lip and mesodermal dysgenesis of the anterior segment of the left eye. Thus it is difficult to claim no relation to Pai syndrome. In patient in this study, there was no mass protruding through the nostril, and the mass was covered with normal nasal mucosa. There were no accompanying pathological findings, thus, the subject was considered to have pure nasal and pericallosal lipoma. We can say that nasal septal fibrolipoma and an accompanying intracranial lipoma can develop on its own without being a part of a syndrome. This is contrary to the previous reports [7].

The classic treatment for lipomas is surgical excision with capsular dissection. Upon learning of the diagnosis of lipoma, it was believed that excision would be simple, due to its well circumscribed capsule. Yet during the operation, this was not the case, because, the lateral border of the mass was adhered to the submucosal tissues of the nasal septum, thus a mucosal part of nasal septum was excised, due to its lateral location in relation to the mass which was unexpected. This may be the future of nasal submucosal fibrolipoma, but it is quite assertive to say so. This point should be highlighted for surgeons who will encounter such septal fibrolipomas.

## Summary

As an uncommon variant of a common neoplasm, fibrolipoma can develop in the nasal septum. This case report is the first presentation of a fibrolipoma in the nasal septum, and discusses the difficulties encountered in surgery. It should be kept in mind that an accompanying intracranial lipoma can also exist, if the patient has symptoms such as seizure, dementia, hemiparesis, or raised intracranial pressure findings.

## Consent

Written informed consent was obtained from the patient for publication of this Case report and any accompanying images.

## Competing interests

The authors declare that they have no competing interests.

## Authors’ contributions

The author MO has made the study design and concept, drafted the manuscript, and made critical review. KI and AK have obtained the data and figures, and drafted the manuscript and references. MI has made a critical review for the manuscript and added comments to discussion. All authors read and approved the final manuscript.
